# Medicine in Buffalo during 1898

**Published:** 1899-01

**Authors:** 


**Monthly Review of Medicine and Surgery.**

*EDITORS:*

THOMAS LOTHROP, M. D. - WM. WARREN POTTER, M. D.

All communications, whether of a literary or business nature, should be addressed to the managing editor: 284 FRANKLIN STREET, BUFFALO, N. Y.

VOL. XXXVIII. JANUARY, 1899. No. 6.

### MEDICINE IN BUFFALO DURING 1898.

AT THE beginning of a new year we present, according to custom, a synopsis of medical events that have taken place in this region during the year just closed. While it cannot be stated that the year 1898 has contributed in this city any startling facts to the sum of our knowledge, yet it has been a period of earnest work that has wrought reasonable results as well as some progress in scientific medicine.

The medical societies claim first attention, and these seem to have kept their accustomed pace and to have at least done creditable work. The Medical Society of the County of Erie held its regular meetings, as well as a number of special sessions. At the annual meeting, held Tuesday, January 11, 1898, at which the president, Dr. Lucien Howe, took the chair, the following-named physicians were admitted to membership: Renwick R. Ross, Henry M. Reinhardt, Julius Ullman, Lorenzo Burrows, Katharine S. Munhall, Mary M. Huntley, Edward M. Dooley, William Preiss and D. J. Constantine. The subject of the president's address was Reasons for a law making Credé's method for the prevention of ophthalmia of infancy obligatory in public institutions. A committee was appointed, the president to be chairman, to present the subject to the legislature for action. The society authorised the censors to employ special counsel to prosecute illegal practitioners of medicine. A memorial of Dr. Wm. S. Tremaine was adopted. The scientific program consisted of a paper by Dr. J. G. Thompson, on Colles's fracture; one by Dr. F. H. Stanbro, entitled, Some of the diarrheal diseases of children, one by Dr. W. G. Bissell, on Formaldehyde disinfection, and a discourse by Mr. G. E. Gordon, of Boston, on Modified milk. The society elected the following-named officers to serve during 1898: President, Lucien Howe; vice-president, J. B. Coakley; secretary, Franklin C. Gram; treasurer, Edward Clark; librarian, Wm. C. Callanan. Censors, J. B. Coakley, C. E. Congdon, Thomas F. Dwyer, Irving W. Potter and J. F. Krug. Delegates to the medical society of the State of New York, Herbert U. Williams, Arthur W. Hurd, Peter W. Van Peyma, Thomas B. Carpenter, Grover W. Wende, Eugene A. Smith, Earl P. Lothrop, of Buffalo, and J. G. Thompson, of Angola.

The semi-annual meeting was held Tuesday, June 14th, at which time the following new members were admitted: L. E. McC. Pomeroy, Francis E. Fronczak, Carro Julia Cummings, John J. Finnerty, F. W. McGuire and John H. Daniels. Dr. Roswell Park read a paper entitled, Gunshot wounds made by modern missiles and their treatment.

Next in order came the Buffalo Academy of Medicine with its usual program. During the year several physicians from other cities have read papers before the different sections, as follows: Tuesday, January 25th, Dr. James F. W. Ross, of Toronto, before the section on gynecology—subject, Torn and punctured wounds of the uterus and vagina; symptoms, prognosis and treatment. Tuesday, February 1st, Dr. Dudley P. Allen, Cleveland—subject, Technique of hysterectomy, both vaginal and abdominal. Tuesday, February 15th, Prof. Ira Van Gieson, director pathologic institute, of the New York State Commission in Lunacy—subject, Toxic degeneration of the nervous system. Tuesday, March 8th, Dr. C. F. Hoover, Cleveland—subject, Cardio-pulmonary murmurs. Tuesday, March 15th, Dr. L. A. Weigel, Rochester — subject, Anatomy and mechanism of the foot, considered with reference to certain abnormal conditions. Tuesday, April 5th, Dr. H. R. Gaylord, Philadelphia—subject, Lantern exhibitions of pathologic specimens; also stereopticon views of diseases of the heart. Tuesday, April 19th, Miss Mary Forster, of Newnham College, England—subject, Scientific choice of fabrics for underwear. Tuesday, June 28th, Dr. Joseph Price, Philadelphia—subject, Discussion and diagnosis of suppurative forms of intrapelvic disease and their surgical management. Monday, July 11th, Dr. Jokichi Tokamine, of Tokio, Japan—subject, Production of diastase and of an alcoholic ferment from fungi. Tuesday, September 20th, Dr. M. V. Ball, Warren, Pa.—subject, Convicts' skulls and brains. Tuesday, November 8th, Dr. L. Pierce Clark, Sonyea—subject, Hypertrophic forms of infantile palsy. Tuesday, November 15th, Dr. Charles D. Aaron, Detroit—subject, Carcinoma of the duodenum. Tuesday, December 6th, Dr. Nathan Jacobson, Syracuse—subject, Curability of cancer. Tuesday, December 20th, Dr. Richard C. Cabot, of Boston—subject, Tests of renal insufficiency by methylene blue and iodine. The annual meeting of the Academy was held Tuesday, June 14, 1898, when the following-named officers were elected for the session of 1898-1899: President, Dr. Roswell Park; secretary, Dr. Thomas F. Dwyer; treasurer, Dr. Charles S. Jewett; trustees, Drs. B. G. Long (3 years), M. Hartwig (2 years), DeLancey Rochester (1 year). Section on medicine: chairman, Dr. William G. Ring; secretary, Dr. Fridolin Thoma. Section on surgery: chairman, Dr. John Parmenter; secretary, Dr. J. Henry Dowd. Section on obstetrics: chairman, Dr. William G. Taylor; secretary, Dr. N. Gorham Russell. Section on pathology: chairman, Dr. A. L. Benedict; secretary, Dr. A. G. Bennett. Council: the above-named officers and the president of the Academy of the previous year, Dr. Lucien Howe.

The private medical societies are as follows: The Medical Club, The Medical Union, The Roswell Park Medical Club, The Physician's Club, The Esculapian Club and the Microscopical society. These are all said to have enjoyed prosperity and their work is reported to have been satisfactory in quality.

The American Electro-therapeutic Association held its annual meeting in Buffalo, September 15, 16 and 17, 1898, under the presidency of Dr. C. R. Dickson, of Toronto. The attendance was good and the papers were of interest. The local committee of arrangements, under the leadership of Dr. Ernest Wende, provided liberally for the entertainment of the visitors. Dr. Wende was elected a vice-president of the association.

The University of Buffalo observed the fifty-second anniversary of its Medical Department with its annual commencement exercises on Tuesday, April 26, 1898, at which 166 graduates in the three departments of medicine, pharmacy and dentistry received their diplomas. Dr. D. A. Currie, '64, of Englewood, N. J., president of the Alumni Association, presided over its deliberations and delivered an address on Living and dying, their physics and psychics. Dr. Daniel Lewis, of New York, president of the state board of health, presented the subject of cancer in an address, entitled, The pre-cancerous stage. Dr. A. M. Houghton, of Detroit, read a paper on Antitoxic serum. Dr. Frank A. Jones, of Rochester, read one on Diphtheria, and others discussed the several subjects presented.

At Music Hall, in the evening, the Rev. O. P. Gifford addressed the graduated classes and afterward the annual banquet was attended at the Ellicott Club.

The University of Niagara observed the thirteenth annual convocation of its medical department, Wednesday, May 11, 1898, at which ten graduates received their diplomas. Dr. Frank W. Malony, of Rochester, was president of the Alumni Association and conducted its proceedings. Rheumatism was the subject of his address. Dr. M. Hartwig, of Buffalo, read a paper on Incipient orthopedic cases; Dr. William G. Ring read one on Movable kidney; Dr. L. Pierce Clark, of Sonyea, presented the subject of the Pathogeny of an epileptic fit, and Dr. L. J. McAdam, of Buffalo, made some remarks on Appendicitis, with cases. At Concert Hall, in the evening, Rev. P. McHale, president and vice-chancellor of the university, addressed the graduates, and the annual banquet was afterward served at the Genesee Hotel.

The announcement was first publicly made June 21, 1898, that the two medical schools—Medical Department of the University of Buffalo and Niagara University Medical College—had consolidated. The faculties were amalgamated and henceforth Buffalo University Medical College will occupy the field alone in this city.

A state pathologic laboratory has been established at Buffalo during the past year, the special purpose of which is investigation into the causes and cure of cancer. The legislature appropriated $10,000 for its maintenance and Dr. Roswell Park, through whose suggestion we believe this was done, has been placed in charge. It is in working order in rooms set apart for it in the Medical College building.

The Sanitory Club has done some excellent work during the year, but its meeting of December 14th attracted special attention on account of the general interest manifested in the subject which it considered—namely, Hygienic military camps. A number of regular army and volunteer officers were announced to speak, and these, together with several physicians and engineers from civil life, who discussed various phases of the subject, served to make an interesting and instructive program.

The literature of the year consists of the following: A Manual of Bacteriology, by Herbert U. Williams, professor of pathology and bacteriology at Buffalo University Medical College, lately issued from the press of P. Blakiston's Son & Company, of Philadelphia. Dr. Williams has also prepared a revision of Miller's Histology for

Students, which appeared in September from the press of William Wood & Co., of New York. These two works constitute an appreciable and important addition to the literature of the subjects with which they deal.

Among the dead of the year were some of our prominent physicians. The list is as follows :

Dr. William Scott Tremaine, January 9, 1898, aged 59 years ; Dr. Michael Talbot, of Niagara Falls, January 26, 1898, aged 51 years ; Dr. John Cronyn, February 11, 1898, aged 72 years ; Dr. Alonzo S. Hinkley, February 25, 1898, aged 75 years ; Dr. J. J. Towle, of Jamestown, March 11, 1898, aged 61 years ; Dr. John J. Cullinane, March 31, 1898, aged 31 years ; Dr. A. P. Southwick, June 5, 1898, aged— ; Dr. C. C. Johnson, of Gowanda, June 7, 1898, aged— ; Dr. John Boardman, July 9, 1898, aged 70 years ; Dr. Joseph Haberstro, July 12, 1898, aged 44 years ; Dr. Harry A. Powers, October 24, 1898, aged 28 years ; Dr. A. E. Willard, of Friendship, November 20, 1898, aged 67 years ; Dr. E. M. Stillman, of Andover, December 5, 1898, aged 57 years ; Dr. Charles S. Hoyt, of Canandaigua, N. Y., December 13, 1898, aged 76 years, and Robert C. Turner, medical student, U. of B. '01, November, 1898.

The recent war with Spain afforded an opportunity for a number of Buffalo physicians to enter the volunteer army, but we are unable to complete the list at this writing. We expect, however, to do so soon in the medical history of Erie County, now publishing in the JOURNAL as a serial.

